# Impact of SMOFlipid emulsion integration in total parenteral nutrition on inpatient outcomes and economic burden for preterm neonates: a retrospective cohort study

**DOI:** 10.1186/s12887-025-05406-0

**Published:** 2025-02-20

**Authors:** Mohamed Emad Eldin Refaay, Omaima Gaber Yassine, Ghada Ahmed Abu-Sheasha, Adel Zaki

**Affiliations:** Department of Biomedical Informatics and Medical Statistics, Medical Research Institute, Alexandria University, 165 El-Horreya Avenue, El-Hadara, POB: 21561, Alexandria, Egypt

**Keywords:** Preterm newborns, Parenteral nutrition, SMOFlipid, Neonatal sepsis, Healthcare economics

## Abstract

**Introduction:**

The use of lipid emulsion in TPN for preterm infants can affect clinical outcomes.

**Objective:**

To assess the potential beneficial health outcomes and economic impact of including SMOFlipid emulsion in TPN for preterm infants, specifically focusing on the incidence of neonatal sepsis, LOS in the NICU, and the associated economic implications from the perspective of healthcare providers in Egypt.

**Methods:**

A record-based retrospective cohort study was conducted in 2021. We collected data from the neonatal intensive care unit (NICU) of Alexandria University Pediatric Hospital, Egypt, for newborns who received TPN. The collected data included gestational age, birth weight, sex, diagnosis of sepsis, LOS in the NICU and type of TPN. Our sample consisted of 103 newborns who received TPN with SMOFlipid and 104 newborns who received TPN without SMOFlipid. Using the propensity score, the study groups’ baseline characteristics were balanced. Odds ratios were estimated using logistic regression, and the statistical significance was set at < 0.05.

**Results:**

Compared to TPN without lipids, the use of SMOFlipid was associated with an observed reduction in the risk of sepsis (OR = 0.64, 95% CI: 0.272–1.51), as well as a difference in the mean reduction in hospital stay (4.12 days, *p* = 0.08). Furthermore, using SMOFlipid was associated with a significant mean reduction in cost of 6,396 EGP (95% CI: 1,491–11,546 EGP).

**Conclusion:**

The integration of SMOFlipid into TPN for preterm infants is associated with decreased incidence of sepsis, reduced NICU stay, and significant cost savings, warranting consideration for standard care practices.

## Background

Preterm infants, particularly those with a very low birth weight (less than 1500 g), rely entirely on parenteral nutrition to meet their nutritional requirements [1].

Total Parenteral Nutrition (TPN) can be life-saving, as it can provide optimal nutrition essential for the growth and development of preterm infants. It is an intravenous method providing a balanced mix of amino acids, dextrose for energy, and an intravenous fat emulsion for essential fatty acids, electrolytes, vitamins, and minerals. It is tailored to meet individual nutritional needs when oral or enteral feeding is not feasible. Electrolytes, such as sodium, potassium, chloride, calcium, phosphate, and magnesium, are carefully balanced within TPN solutions to maintain the crucial electrolyte equilibrium essential for bodily functions [2, 3].

The imbalance of adequate nutrients in TPN significantly increases hospital length of stay (LOS), nosocomial infections, and mortality [4]. This can increase the cost of healthcare for preterm infants [5].

Certain nutritional lipid components, such as long-chain ω-3 polyunsaturated fatty acids (LC n-3 PUFA), are crucial to reach goal energy intake and have a significant role in early retinal and brain development and anti-inflammatory effects. The choice of the appropriate lipid emulsion has an impact on clinical outcomes in preterm infants [6, 7].

Traditional lipids include conventional soy-based lipid emulsions (intralipid emulsions), which are rich in linoleic acid and arachidonic acid (ARA) (ω-6 PUFA). depresses cell-mediated immunity, causing impaired immunity [8, 9], and promoting an inflammatory response [10]. Due to its nutritional value and iso-osmotic (mean pH: 7.5) characteristics, bacterial contamination is another crucial safety concern.

Despite multiple therapies, neonatal sepsis continues to be a substantial cause of morbidity and mortality in newborns [11].

In Egypt, the reported incidence rate of sepsis in the Neonatal Intensive Care Unit (NICU) is approximately 33% [12, 13]. Many studies have found an association between bacteremia outbreaks in the NICU and intravenous lipid emulsion, which consequently elevates the risk of infections, mortality rates, and extends the duration of hospital stays with high costs associated with the total treatment [14–16]. This dilemma places healthcare providers in a precarious position: the imperative to administer lipid solutions as a component of TPN to support the nutritional needs of preterm infants starkly contrasts with the potential adverse health outcomes associated with traditional lipid emulsions.

An alternative lipid emulsion, fish oil (FO)-containing lipid emulsions such as SMOFlipid (Fresenius Kabi), is a mixed-composite lipid emulsion. It differs from standard emulsions by increasing omega-3 (α-linolenic acid, eicosapentaenoic acid (EPA), and docosahexaenoic acid (DHA)), α-tocopherol, oleic acid (mono-unsaturated omega-9 fatty acid), and medium chain triglycerides (MCT) [17] while decreasing the content of omega-6 (Linoleic acid) [17–20].

Using SMOFlipid led to improved metabolism with a reversed energy deficit. It modulates oxidative stress by reducing the impact of lipid peroxidation on oxidative stress. It increased antioxidants, leading to enhanced immune system strength and reduced immunosuppression. In preterm infants with preexisting inflammation, it prevented the occurrence of hyperinflammation [21]. Although there is evidence of the beneficial effects of lipid emulsions enriched in PUFA n-3, MCT, and LCT, there is insufficient information regarding their cost-effectiveness [22]. Using SMOFlipid emulsion in preterm infants may have a cost-minimization effect by reducing the incidence of sepsis, a serious and costly complication [23]. However, the utility of this nutritional regimen has not yet been well studied in developing countries with limited resources.

Considering these challenges, our study aims to elucidate the potential beneficial health outcomes and economic impact of including SMOFlipid emulsion in TPN for preterm infants, specifically focusing on the incidence of neonatal sepsis, LOS in the NICU, and the associated economic implications from the perspective of healthcare providers in Egypt.

## Materials and methods

### Study design

A record-based retrospective cohort study.

### Study setting

We conducted the study in the NICUs of Alexandria University pediatric hospital in Egypt, a single-center institution with two NICUs. Both units followed the same protocols and policies; however, one unit used SMOFlipid, while the other did not use any lipid emulsions.

### Study population

The study included preterm infants with a gestational age ranging from 26 to 35 weeks who received TPN for seven days or more. The components used in TPN were consistent across all infants. These infants were admitted to the NICU in 2021 and were free from sepsis at the time of admission. Preterm infants were classified into two groups:

#### Group 1

Preterm infants received total parenteral nutrition with SMOFlipid emulsion.

#### Group 2

Preterm infants received total parenteral nutrition without lipid emulsion.

### Exclusion criteria

- Preterm infants who received blood transfusions or preterm infants who received any medications were associated with either an increased or decreased risk of sepsis. Specifically, medications such as immunosuppressants or corticosteroids can increase the risk of sepsis, whereas immunomodulatory drugs can decrease it.
- Preterm infants who received milk feeding or any alternative lipid sources other than SMOFlipid.

### Pilot test

A pilot test was conducted on 40 preterm infants to ensure the feasibility of the study, refine our research questions and hypotheses, and calculate the required sample size.

### Sample size calculation

Based on our pilot study, the incidence of sepsis in preterm infants who did not receive lipid emulsion was 15% higher than that among preterm infants who received SMOFlipid emulsion. In each group, a sample size of 100 preterm infants had 80% power to detect a 15% difference in the primary outcome between the two groups with a 95% confidence level. **(Epi Info ™**, **version 7.2.4.0).**

### Data Collection

Patient records were retrieved, and the following data were extracted:

1. Demographic data: gestational age in weeks, sex, and birth weight.
2. Clinical Outcomes: During the study period, a crucial clinical outcome, the occurrence of sepsis in each preterm infant, was closely monitored and documented. All preterm infants had positive blood cultures.
3. TPN Regimen: Information on whether the preterm infants received TPN with or without SMOFlipid emulsion was collected.
4. The costs include the cost of medications and TPN, whether with or without SMOFlipid, the cost of LOS in the NICU, and the cost of culture. All costs are listed in Table 1 during the periods from January 1st, 2021, to January 1st, 2022, in Alexandria, Egypt, in 2022 Egyptian pounds (EGP) and converted to 2022 US dollars (USD), adjusting for inflation and currency differences using the Gross Domestic Product (GDP) deflator index and purchasing power parities, respectively [24]. We only accounted for the direct medical costs of treatment during hospitalization. To maintain precision and clarity, other potential costs beyond this scope were not taken into account.
5. The length of stay (LOS) in the NICU is measured in days.

### Ethical considerations

- Guidelines of the ethical committee of the Medical Research Institute were followed, and data were anonymized to maintain patients’ confidentiality (Serial No. E/C. S/N. T43/2022).
- Permission of Alexandria University hospitals to conduct the study was granted after providing all information about the study purpose and ensuring confidentiality of the patients’ information (Serial No. 0107286).

**Table 1:** Units costs of resources used in treating preterm infants at the NICU during the periods from January 1st, 2021, to January 1st, 2022, Alexandria, Egypt

Varibales		Egyptian pounds (EGP)	US dollars (USD)
Medications per day	Antibiotics	200–500	13–32
	Antifungal	100	7
	Parenteral nutrition	500–550	32–35
	Inotropes Drugs	100	7
Hospitlization in NICU per day		800	51
Culture senistivity		250	16

### Statistical methods

Statistical analysis was performed using the Statistical Package for Social Sciences (SPSS) version 28 and the epiR and ggplot2 R versions 4.0.4 [25]. Data are presented as the mean ± standard deviation (SD), and an independent *t* test was used to compare groups. Categorical variables were described using frequency and percentages, and Pearson’s chi-square test (χ^2^) was used to compare groups. The odds ratios (ORs) of logistic regression analysis were used to assess associations between receiving SMOFlipid, birth weight, gestational age, sex, and the occurrence of sepsis. Statistical significance was set at < 0.05. Additionally, we compared binary and continuous outcomes using logistic and gamma regressions, respectively. The odds ratio (OR), mean difference, and adjusted OR with 95% bootstrap confidence intervals (BaCI) were used to report the results. Due to the equivalence of the outcome among the studied groups, we conducted a cost minimization evaluation by calculating the mean difference in the mean total costs between the two studied groups from the perspective of healthcare providers [26].

### Creating balanced groups using the propensity score

Our observational study’s baseline characteristics were unbalanced when comparing outcomes between the two groups as a result of nonrandomization. To solve this problem, Inverse Propensity Score Weighting (IPSW) was used to equalize pretreatment variations across groups, where scores were estimated using the Generalized Boosted Model (GBM) with 2000 iterations. Sex, gestational age, and birth weight were the covariates considered when calculating the propensity score. We used standardized mean differences (SMDs) to assess the balance of covariates between the two groups [27]. The effective sample size was calculated to estimate the impact of IPSW analysis on the sample size [27]. This was accomplished using the R programming language [25] as well as the Toolkit for Weighting and Analysis of Nonequivalent Groups package (Twang) [27].

## Results

### Baseline characteristics of the studied groups before and after IPSW

According to the order of admission to the NICU, a total of 103 newborns received TPN with SMOFlipid, while 104 received TPN without SMOFlipid in 2021.

**Fig. 1:**
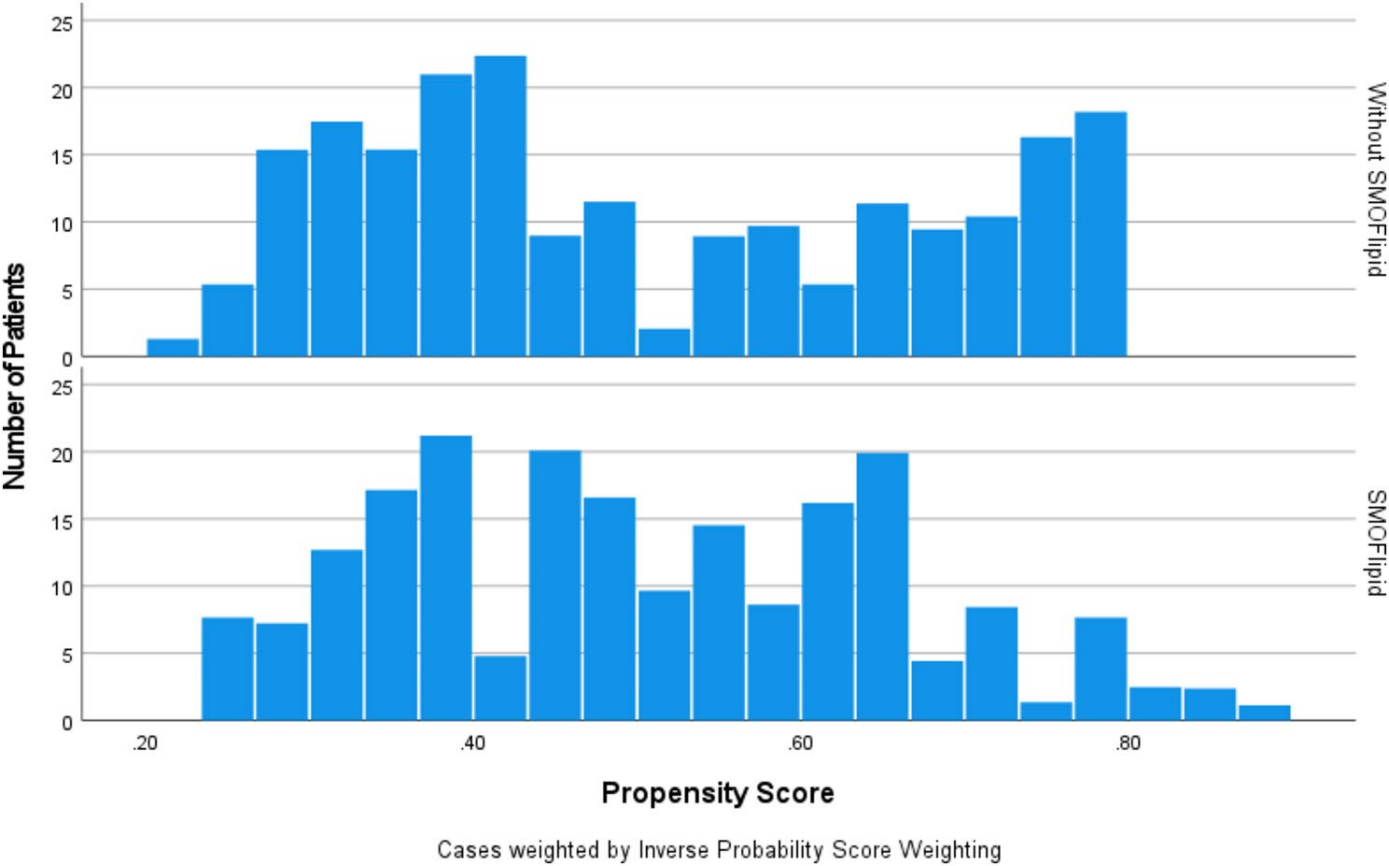
The two groups share substantial overlap in the propensity score

**Fig. 2:**
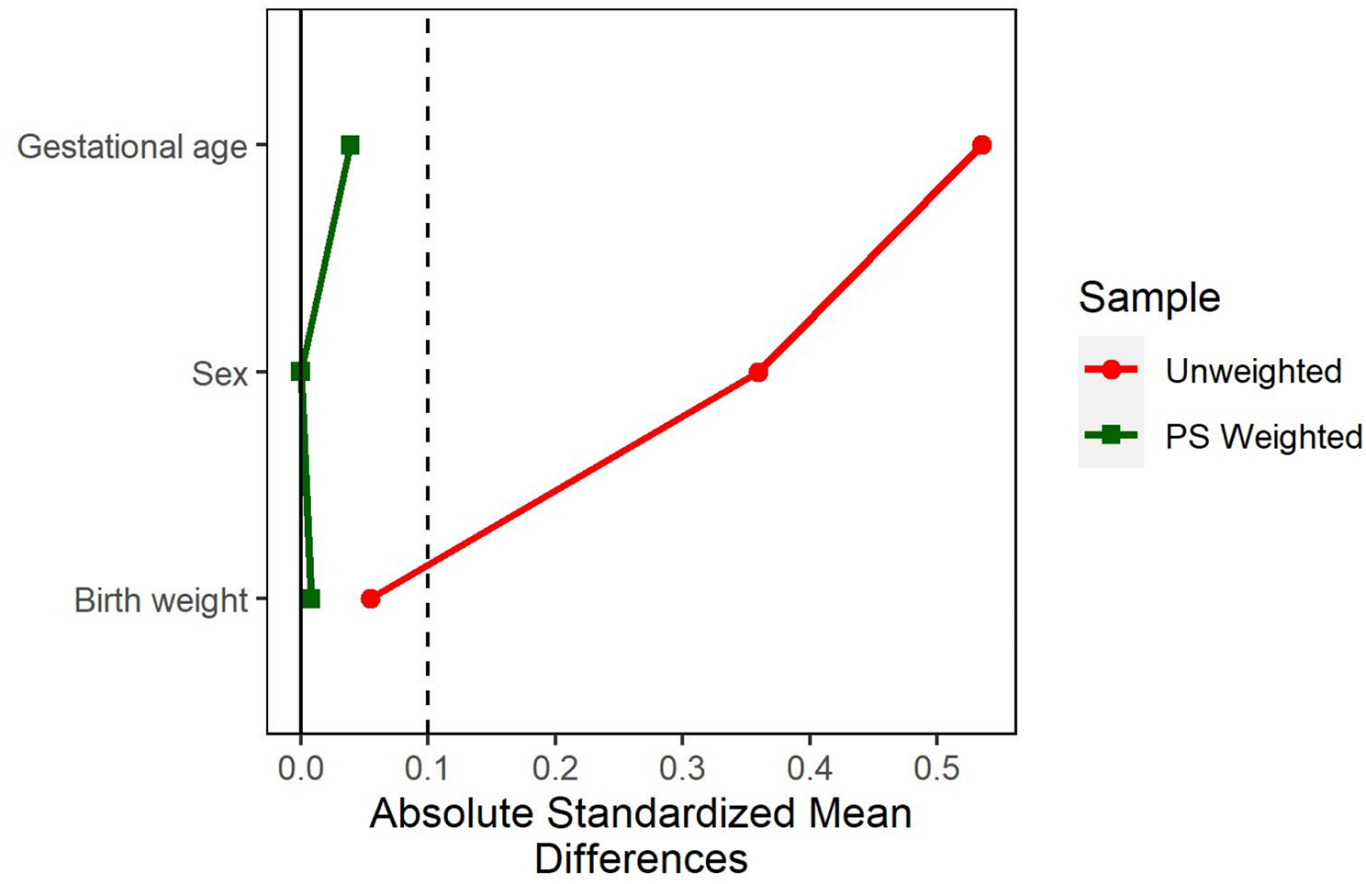
Absolute standardized mean differences in gestational age, sex, and birth weight between both groups before and after IPSW. A vertical line was added to the graph to stand for a standardized difference of 0.1, which is a threshold below which any imbalance is negligible

Table 2 shows a comparison of the baseline characteristics of the two groups. Before adjustment, the mean birth weight was not different between the two groups. In the SMOFlipid group, baseline sex and gestational age were significantly different between newborns who received TPN and those who did not. Figure 1 shows a substantial overlap in scores in both groups. This indicates that the IPSW can be conducted. After IPSW, baseline characteristics were balanced (Fig. 2). For example, females were more common in the TPN with the SMOFlipid group (57%) than in the TPN without the SMOFlipid group (39%). After IPSW, the females were equal in the two studied groups. This suggests an adequate balance between the two groups. All baseline variables showed an *SMD* < 0.1 to improve balance between the two groups (Fig. 2).

**Table 2:** Comparison of baseline characteristics according to the type of total parenteral nutrition among a cohort of preterm infants admitted to NICU of Alexandria university pediatrics hospitals in 2021 before and after IPSW

	Unadjusted							Adjusted by IPSW							
Variables	TPN with SMOFlipid		TPN without SMOFlipid		*P*	aSMD		TPN with SMOFlipid		TPN without SMOFlipid		a *P*	aSMD		
	*n* = 103		*n* = 104					*n* = 103		*n* = 87					
Birth weight (kg)	1.29(0.33)	1.30		(0.035)	0.701		0.06	1.29	(0.33)	1.29	(0.33)	0.962		0.01	
Gestational age (Weeks)	29.41(2.09)		28.29	(1.71)	< 0.001*		0.54	29.41	(2.09)	29.33	(1.97)	0.806		0.04	
Sex (Female)	5957.3%		41	39.4%	< 0.01*		0.36	59	57.3%	50	57.5%	0.999		0.00	

**Sex** is expressed as frequency and percentage. **Birth weight** and **gestational** age are expressed as **mean** and **standard deviation**. ***an*** Adjusted ***P*** value from **IPSW** comparisons (***aSMD*** **< 0.1** indicates that distributions conditioned on the propensity score are balanced)

### Association of SMOFlipid administration with sepsis risk: relative risk analysis

Table 3, which presents a crosstabulation of the occurrence of sepsis in relation to the administration of SMOFlipid, a nutritional intervention after IPSW.

In the SMOFlipid group, the cumulative incidence of sepsis was 12.6%, compared to 18.4% in the non-SMOFlipid group. The calculated RR was 0.69. Thus, preterm with SMOFlipid were less liable to have sepsis than preterm without SMOFlipid. With SMOFlipid, the likelihood of sepsis is reduced by 31%. The calculated AR was 5.8%. Therefore, 5.8% of the risk of sepsis was attributable to the non-introduction of the SMOFlipid emulsion.

The calculated NNT was approximately eighteen. This implies that for every eighteen preterm receiving SMOFlipid, one case of sepsis could be prevented.

**Table 3:** Impact of SMOFlipid on sepsis incidence in preterm infants after IPSW

Using SMOFlipid	Sepsis			
	Yes (%)	No (%)		Total
Yes	13 (12.6%)	90 (87.4%)		103
No	16 (18.4%)	71 (81.6%)		87
Total	29 (15.3%)	161 (84.7%)		190

### Inpatient outcomes difference between the studied groups

Receiving TPN with SMOFlipid in preterm infants was associated with a 36% reduction in the odds of having sepsis compared to those who received TPN without SMOFlipid (OR = 0.64, 95% CI: 0.272–1.51), but the result was not significant (*p* value = 0.305). The gamma regression, where the length of stay was regressed by the treatment group, showed that SMOFlipid was associated with a mean reduction in hospitalization by 4.12 days. The 95% bootstrap confidence interval (BaCI) for this estimate ranged from 0.14 days to 7.58 days, but the result was not significant (*p* value = 0.08). The gamma regression on the treatment group showed that SMOFlipid was associated with a mean reduction in mean total cost of 6,396 EGP (95% CI: 1,491 to 11,546 EGP). A comparison between the studied groups is summarized in Table 4.

**Table 4:** Effect of using TPN with SMOFlipid on sepsis, LOS, and total cost among a cohort of preterm infants after IPSW

Variables	TPN with SMOFlipid	TPN without SMOFlipid	Estimate	95% CI		*P*
				Lower	Upper	
Sepsis	13 (12.6%)	16 (18.3%)	0.64	0.272	1.51	0.305
LOS **(Days)**	29.3 (9.5)	33.4 (20.1)	− 4.12	− 7.58	0.14	0.080
Total cost **(EGP)**	32951 (11249)	39254 (25499)	− 6396	− 11,546	− 1491	0.034*

**Sepsis** is expressed as frequency and percentage. **LOS** and **total cost** are expressed as the **mean** and **standard deviation**. **Estimate**: represent the odds ratio (SMOFlipid vs. No SMOFlipid) for sepsis and the gamma regression coefficient (difference between SMOFlipid and no SMOFlipid) for LOS and total cost

### Measuring the associations between different variables and the occurrence of sepsis

The odds ratio of sepsis occurrence was compared between preterm infants who received TPN with SMOFlipid and those who received TPN without SMOFlipid while controlling for sex, and gestational age (Table 5).

After conducting multivariate analysis, the results show that there was no statistically significant association between preterm infants who received TPN without SMOFlipid and the development of sepsis (*p* value = 0.064), despite a trend towards lower risk in infants receiving SMOFlipid. Additionally, sex did not show a statistically significant association with sepsis development, although males were 1.64 times more likely to develop sepsis compared to females (*p* = 0.196). Gestational age was not significantly associated with sepsis, though an apparent less risk with increase of both.

**Table 5:** Predictors for sepsis in preterm newborns (*N* = 207). Multivariate logistic regression

Variables			
	AOR	95% CI	*P* value
SMOFlipid vs. without SMOFlipid ^**ref1**^	0.48	(0.22–1.04)	0.064
Sex male vs. female ^**ref2**^	1.64	(0.77–3.49)	0.196
Gestational age ^**ref3**^	0.85	(0.69–1.06)	0.144

**AOR**: Adjusted Odd’s Ratio, **CI**: Confidence Interval, **ref**_**1**_: TPN without SMOFlipid, **ref**_**2**_: female **ref**_**3**_: increase 1-week

## Discussion

While lipid emulsions are essential for meeting the nutritional needs of these vulnerable infants, traditional lipid emulsions may be associated with adverse health outcomes [10]. In neonatal care, the decision to use lipid emulsions in TPN for preterm infants presents a complex dilemma, as the results might be influenced by the lack of energy or essential fatty acids (EFA) in the absence of any lipid source [3]. So, the rationale for this comparison stems from the conflict between the potential risks associated with traditional lipid emulsions and the lack of awareness or apprehension among physicians regarding the benefits of alternative lipids such as SMOFlipid.

This study supports the potential benefits of using SMOFlipid in TPN formulations for preterm infants. Although the statistical significance of the reduction in the risk of sepsis attributed to the use of SMOFlipid is borderline (*P* = 0.064), the 52% reduction in the odds of sepsis indicates its potential clinical importance (OR = 0.48, 95% CI: 0.22–1.04). This finding is consistent with previous studies that indicated that SMOFlipid has a protective effect against infection [28–30].

When analyzing the impact of SMOFlipid on hospitalization, the gamma regression analysis indicated that infants receiving TPN with SMOFlipid had a mean reduction in hospitalization by approximately 4.12 days compared to those without SMOFlipid. However, this reduction was not statistically significant (*p* = 0.08). The 95% bootstrap confidence interval for this estimate was wide (0.14 to 7.58 days), indicating variability in the potential reduction of hospital stay associated with SMOFlipid. These results suggest a potential trend toward shorter hospitalization. The reduction in the LOS with the use of SMOFlipid was confirmed in previous studies [28, 31]. Our study also found that males were 1.64 times more likely to develop sepsis compared to females, but this increase in risk was not statistically significant (p value = 0.196). This agrees with a previous study that showed a higher incidence of sepsis in males than in females (1.7:1) [32]. Another study found that male preterm infants are at a higher risk of Sepsis and mortality than female preterm infants [33]. This higher risk of sepsis in male preterm infants was attributed to hormonal, genetic and immunological differences [34].

One of the important findings of our study is that the use of SMOFlipid in TPN was associated with a significant reduction in total cost compared to TPN without SMOFlipid. This result was consistent before and after adjusting for confounding variables using IPSW with bootstrapping. This is an important finding, as reducing healthcare costs while maintaining or improving quality of care is a key objective in healthcare systems worldwide. The gamma regression analysis showed that SMOFlipid was associated with a mean reduction in the mean total cost by EGP 6,396, which is a substantial amount considering the short duration of hospitalization for newborns.

In this study, we used propensity scores and inverse probability weighting to account for confounding variables. This is the first study to look at the use of SMOFlipid in TPN in our health care setting, which is one of its strengths. This statistical method improves the balance between treatment groups. This is particularly relevant for studies conducted in real-world settings, where randomization may not be feasible or ethical. By using inverse probability weighting to adjust for baseline covariates, researchers can obtain more accurate estimates of treatment effects and reduce confounding bias. While the present study provides useful insights into the potential benefits of using SMOFlipid in TPN for preterm infants, it has notable limitations. The research is conducted at a single institution and uses a retrospective design, which may impact the generalizability of the findings and introduce inherent biases. Our research focus on sepsis and NICU LOS is a limitation, as it overlooks other critical outcomes such as the potential protective effects of SMOFlipid against Parenteral Nutrition-Associated Liver Disease (PNALD). Including PNALD and other relevant outcomes in future research is necessary to fully understand the benefits of SMOFlipid in TPN. Despite efforts to address these through advanced statistical techniques, further studies with larger sample sizes, randomized controlled designs, and longer follow-up periods are needed to confirm these findings.

## Conclusion

Our study on health outcomes and potential adverse effects associated with the use of SMOFlipid in TPN for preterm infants highlights a clinically meaningful reduction in sepsis risk and it may be considered a cost-effective.

## Data Availability

The data that support the findings of this study are available from the corresponding author, upon reasonable request.

## Abbreviations

ARA: Arachidonic Acid
CVC: Central Venous Catheter
DHA: Docosahexaenoic Acid
EFA: Essential fatty acids
EPA: Eicosapentaenoic Acid
FO: Fish Oil
GBM: Generalized Boosted Model
IPSW: Inverse propensity score weighting
K–S test: Kolmogorov–Smirnov test
LC-PUFA: The long-chain polyunsaturated fatty acid
LC n-3 PUFA: Long-chain ω-3 polyunsaturated fatty 5 acids
LCT: Long-chain Triglyceride
LOS: Length of Stay
MCT: Medium-chain Triglyceride
NICU: Neonatal Intensive Care Unit
ORs: Odds Ratio
PH: Power of Hydrogen
PNALD: Parenteral Nutrition-Associated Liver Disease
SD: Standard Deviation
SPSS: Statistical Package for Social Sciences
TPN: Total Parenteral Nutrition
